# Altered emotional interference processing in the amygdala and insula in women with Post-Traumatic Stress Disorder^☆^^☆☆^

**DOI:** 10.1016/j.nicl.2012.11.003

**Published:** 2012-11-13

**Authors:** Steven E. Bruce, Katherine R. Buchholz, Wilson J. Brown, Laura Yan, Anthony Durbin, Yvette I. Sheline

**Affiliations:** a Department of Psychology, University of Missouri, St. Louis, United States; b Department of Psychiatry, Washington University in St. Louis, St. Louis, Missouri, United States; c Department of Neurology, Washington University in St. Louis, St. Louis, Missouri, United States; d Department of Radiolology, Washington University in St. Louis, St. Louis, Missouri, United States

**Keywords:** PTSD, fMRI, Trauma, Amygdala, Insula, Default mode network

## Abstract

**Background:**

Post-Traumatic Stress Disorder (PTSD) is characterized by distinct behavioral and physiological changes. Given the significant impairments related to PTSD, examination of the biological underpinnings is crucial to the development of theoretical models and improved treatments of PTSD.

**Methods:**

We used an attentional interference task using emotional distracters to test for top-down versus bottom-up dysfunction in the interaction of cognitive-control circuitry and emotion-processing circuitry. A total of 32 women with PTSD (based on an interpersonal trauma) and 21 matched controls were tested. Event-related functional magnetic resonance imaging was carried out as participants directly attended to, or attempted to ignore, fear-related stimuli.

**Results:**

Compared to controls, patients with PTSD showed hyperactivity in several brain regions, including the amygdala, insula, as well as dorsal lateral and ventral PFC regions.

**Conclusions:**

These results are consistent with previous studies that have higher amygdala and insular activation in PTSD subjects. However, inhibition of suppression of PFC regions is inconsistent with the fear circuitry model hypothesized by prior research. We suggest that the specific emotional conflict task used appears to target implicit or automatic emotional regulation instead of explicit or effortful emotional regulation. This is particularly relevant as it posited that emotional regulatory difficulties in anxiety disorders such as PTSD appear to occur in implicit forms of emotion regulation.

## Highlights

► We use an emotional interference task to examine brain activity in women with PTSD. ► Significant hyperactivity was found in amygdala and insula in PTSD subjects. ► Hyperactivity in PFC regions were inconsistent with the fear circuitry hypothesis. ► Findings suggest PTSD subjects fail to normally down-regulate activity in the DMN.

## 1 Introduction

Posttraumatic stress disorder (PTSD) is a prevalent and chronic anxiety disorder with distinct behavioral and physiological changes. It is associated with persistent re-experiencing, avoidance of trauma reminders, hypervigilance and exaggerated startle response. PTSD results in significant impairment across a multitude of domains, including social, occupational, recreational, and sexual functioning. Further, PTSD has been found to be one of the highest predictors of subsequent suicidal planning or attempts (Kessler, 2000; Bruce et al., 2001; Sareen et al., 2005; Nock et al., 2010). Thus, the public health implications are substantial. Sexual and physical violence are serious problems that affect millions of individuals every year. An estimated 17% of women have reported being sexually assaulted in their lifetime (Tjaden and Thoennes, 2000) and among a national sample of college students, 20%–25% of women reported experiencing completed or attempted rape (Fisher et al., 2000). The consequences of being a victim of sexual and physical violence are enormous and often have serious and long-term effects. Given the importance of PTSD, considerable effort is being directed toward better understanding the biological underpinnings.

Previous studies examining the neurocircuitry of PTSD have connected abnormalities in several brain regions including the amygdala and insula as well as the ventral medial and dorsal lateral prefrontal cortex (vmPFC, dlPFC), which include the anterior cingulate cortex [ACC] and ventral medial frontal gyrus. A“fear circuitry” model of PTSD posits that the amygdala and insula are hyperresponsive, thereby increasing fear and anxiety responses. Conversely, the ventral mPFC is hypothesized to be hyporesponsive and as a result, fails to inhibit the amygdala. Moreover, recent research has found that the dorsal anterior cingulate cortex (dACC) is hyperresponsive in PTSD (Hughes and Shin., 2011; Shin et al., 2007).

There have been numerous studies implicating amygdalar hyperactivity in PTSD using a variety of trauma-related stimuli, including imagery as well as trauma-related words and sounds (Liberzon et al., 1999; Rauch et al., 2000; Pissiota et al., 2002; Hendler et al., 2003; Vermetten and Bremner, 2003; Protopopescu et al., 2005; Shin et al., 2005; Bryant et al., 2008a, 2008b; Felmingham et al., 2010). Heightened amygdala activity in response to non-trauma related emotional stimuli such as fearful faces has also been found (Rauch et al., 2000; Bryant et al., 2008a, 2008b; Felmingham et al., 2010). For example, in a study examining veterans with PTSD, Rauch et al. (2000) found increased hyper amygdala activation in response to masked fearful faces compared to masked happy faces. However, other studies have failed to show overactivation of the amygdala in response to emotional stimuli, including a recent study by Kim et al. (2008). In this study, twelve victims of a subway fire in South Korea were presented with an emotional conflict task comprised of emotional facial expressions paired with pictures of houses. Compared to a trauma-exposed healthy control group who also experienced the same subway fire, the authors did not find evidence of increased amygdala activity in the PTSD group. This is particularly noteworthy as the paradigm is similar to that of the current study.

Though the role of the amygdala in PTSD has been frequently cited, until recently the insula has been less of a focus. Given that the insula is heavily connected with the amygdala and regulates the autonomic nervous system, it is critical to examine the role it may play in fear conditioning and PTSD. Though the amygdala is a key component of the fear response, the insula has been found to be involved in more generalized anxiety responses, including interoceptive and anticipatory anxiety (Schunck et al., 2008; Lovero et al., 2009). Thus, the combination of the amygdala and insula appears to have a unique and complementary role in limbic and emotional processing (Craig, 2009). Examination of PTSD studies of the insula confirms increased activation of the right middle insula (Strigo et al., 2010) in a sample of women with intimate partner violence-related PTSD. Other studies have also shown increased activation of the insula (Lanius et al., 2007; Lindauer et al., 2008). Using script-driven imagery, Lindauer et al. (2008) found higher activation in the right insula in PTSD subjects compared with trauma-exposed controls Findings of higher insula activation also extend to emotional, trauma-unrelated stimuli (Simmons et al, 2008, Fonzo et al, 2010).

The primary aim of the current study was to use fMRI in combination with performance of a cognitive emotion-processing task (or emotional interference task) to examine amygdala and insula activation in women who meet criteria for PTSD due to an interpersonal trauma compared with healthy controls. Further, given results of prior research showing reduced VMPFC activity in PTSD, in concert with amygdala hyperreactivity, we also examined task-related activation in the medial prefrontal cortex and its relationship with amygdala and insula activity. The emotional-interference task was specifically chosen to discern if there were differences in amygdala and insula activity while consciously attending to (or ignoring) emotional stimuli while engaged in a cognitive task. Using an implicit as opposed to an explicit emotional regulation task determines whether it is necessary that PTSD patients attend to fearful stimuli, or if the mere presence of fearful stimuli (when the subject is trying to ignore the stimulus) is associated with increased amygdala and insula activation.

## 2 Methods

### 2.1 Participants

Participants were 32 females with PTSD (mean age: 31.5 years (*SD* 9.2), mean education: 15.4 years (*SD* 3.0)), and 21 demographically matched trauma-unexposed female controls (mean age: 30.6 years (*SD* 7.6), mean education: 16.4 years (*SD* 2.7)). The PTSD group included 18 Caucasians, 14 African Americans; the control group included 16 Caucasians, 4 African Americans, and one Asian. No significant demographic differences (age, ethnicity, education level) were found between the PTSD and matched control groups.

To be included in the study, participants had to be right-handed, English-speaking females, aged 18–55, of any race, with the capacity to give informed consent and to follow study procedures. Candidates for the PTSD group must have been exposed to an interpersonal trauma, and meet current DSM-IV-TR criteria for Post-Traumatic Stress Disorder based on the Clinician-Administered PTSD Scale (CAPS; (Blake et al., 1995, 1998). The CAPS is a 30-item structured interview that corresponds to the DSM-IV criteria for PTSD. Based on a previous study of childhood sexual abuse in women (Orr et al., 1997) participants must have had a CAPS score greater than 45. In addition, participants must have met the original scoring criteria proposed by Blake et al. (1995) in which a PTSD symptom is considered present if the frequency of the CAPS item is rated as 1 or higher and the intensity is rated at a 2 or higher. The average CAPS score for the 32 participants with PTSD was 66.18 (*SD* = 13.78).

Participants were excluded from the study if (1) they had been diagnosed with neurological disorders such as dementia, stroke, encephalopathy Parkinson's Disease, brain tumors, multiple sclerosis, or seizure disorder; (2) they showed any signs of current alcohol or substance abuse disorder, schizophrenia or other psychotic disorder, bipolar disorder, or current obsessive-compulsive disorder (OCD); (3) they displayed active suicidality or presented a current suicidal risk, as determined by the investigator, or (4) they were currently being treated with psychotropic drugs or drugs that affect the CNS such as beta-blockers, mood stabilizers, antipsychotics or other antidepressants. No subject was included in the study unless they had been off all psychotropics for at least three weeks, (or in the case of fluoxetine, for five weeks). In addition, participants were excluded if they had significant handicaps that would interfere with testing procedures (e.g., uncorrected visual or hearing loss, and mental retardation), if they had implanted devices (such as a pacemaker) or other metallic objects in the body that serve as contraindications for MRI scanning, or if they had any other factor that in the investigator's judgment might affect patient safety or compliance (e.g. distance greater than 100 miles from clinic).

All participants provided written informed consent in accordance with criteria established by both the University of Missouri-St. Louis and Washington University's Human Subjects Committees. Participants were paid $60.00 for their participation if they completed the entire scanning session that day (or $10 per hour otherwise).

### 2.2 Procedure

The emotional interference experiment was carried out as part of a study that included several phases. An initial day of assessment (including psychiatric evaluation as well as and mood/personality questionnaires) included no scanning. Scanning for the emotional interference task occurred on a second day and was always carried out before other tasks. The entire scan took approximately 1.5 h, with the emotional interference task lasting approximately 30 min. At the beginning of the emotional interference scan, participants were instructed on how to do the task, to emphasize speed and not worry about mistakes. They were given practice trials inside the scanner, using neutral faces only.

**Fig. 1:**
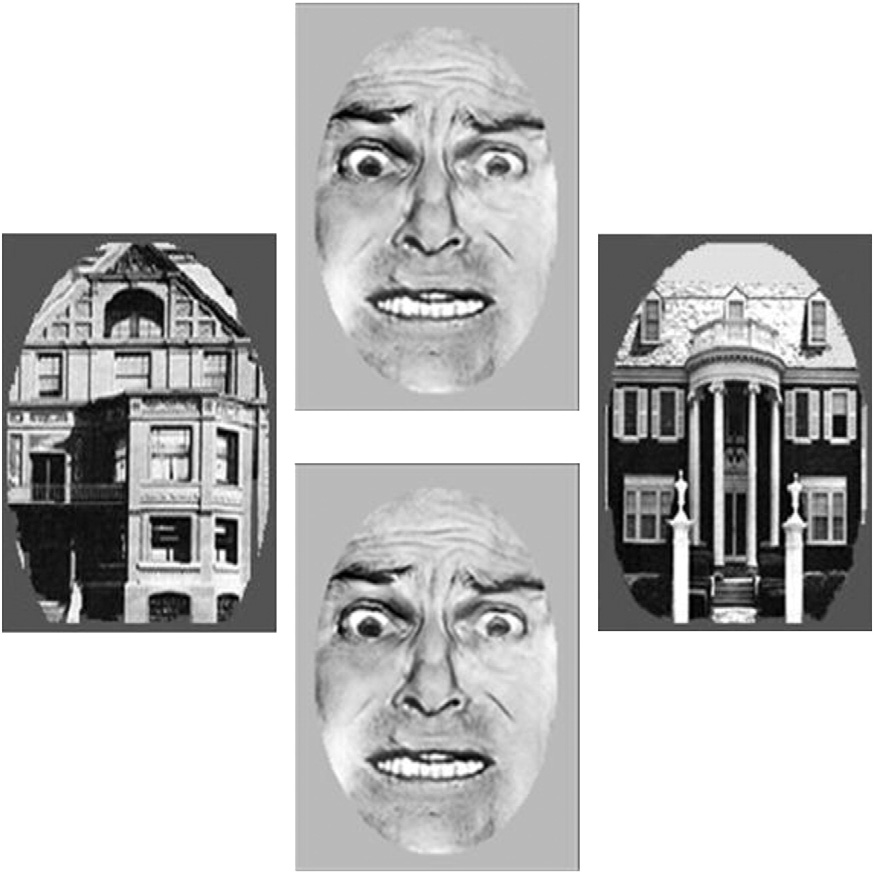
Example of a stimulus screen used in the emotional conflict task.

The emotional-interference task (Vuilleumier et al., 2001; Bishop et al., 2004; Fales et al., 2008) presented participants with a pair of houses and a pair of faces in each trial (Fig. 1). An implicit emotional regulation task examines the use of non-conscious cognitive control to regulate the conflict between a cognitive and an emotional task with distractors (Etkin and Schatzberg, 2011), In this study, one pair was arranged horizontally and the other vertically around a central fixation cross. Participants were instructed to fixate on the cross and attend to the horizontal or vertical axis for a given block (4 blocks total, counterbalanced order). Positioning of face-pairs or house-pairs was random. For each trial, the task was to tell whether the two items in the target axis were the same or different. Participants responded by button-press on a fiber optic response box that interfaced with PsyScope (Cohen et al., 1993). Each block contained 13 trials for each attention × emotion condition, pseudo-randomly interleaved throughout the block (52 trials total per block). Thus trial types were: attend-fearful-faces, attend-neutral-faces, ignore-fearful-faces (attend-houses), and ignore-neutral-faces (attend-houses). For each trial, the two faces displayed were either both neutral or both fearful, with the two expression types occurring equally often in a block. Each trial lasted 3200 ms, starting with a fixation (displayed for 1000 ms), after which the four stimuli appeared for 250 ms. Participants had 2200 ms to make a response. An intertribal interval (ITI) then took place that varied randomly between five possible lengths (2150, 4660, 7170, 9680, or 12,190 ms).

### 2.3 fMRI imaging and analysis

#### 2.3.1 Image acquisition

fMRI images were collected on a Siemens 3 T TrioTim MRI scanner (Erlangen, Germany). The protocol included localizer images, a high-resolution structural image (magnetization prepared rapid gradient echo (MPRAGE)), and a series of functional images. The structural images were acquired with 1 × 1 × 1 resolution using a sagittal 3-D T1-weighted sequence with repetition time (TR) of 2.4 s, time-to-echo (TE) of 3.13 ms, flip angle = 8°, and inversion time (TI) of 1000 ms. Functional images were collected using an asymmetric spin-echo echo-planar sequence TR = 2.2 s, TE = 27 ms, flip angle = 90° and field of view (FOV) of 256 cm. One acquisition consisted of 36 transverse slices, 4 mm thick (no gap), and with an in-plane resolution of 4 × 4 mm. Each functional run began with four volume images that were not analyzed, followed by 180 acquisitions for the paradigm.

#### 2.3.2 Image analysis

The functional imaging data were preprocessed to correct for asynchronous slice acquisition and odd/even slice intensity differences caused by interleaving. Following this, the data were rigid body motion corrected (Friston et al., 1996; Snyder, 1996). Atlas transformation (12 parameter affine) of the functional data was computed via the structural images. Our atlas representative target image conforms to the space of (Talairach and Tournoux, 1988), as defined by Lancaster et al. (1995). The final preprocessing step combined motion correction and atlas transformation in one resampling to 3 mm isotropic voxels. Before statistical analysis, the data were smoothed using a Gaussian filter with 9 mm full-width half-maximum.

For each participant, a General Linear Model (GLM) was used to estimate hemodynamic model-independent (Ollinger et al., 2001) event-related responses over 17.6 s (eight frames). Separate regressors were used to estimate responses of four conditions: 1) attend to fearful faces, 2) attend to neutral faces, 3) ignore fearful faces and 4) ignore neutral faces. We computed a response timecourse estimate for each condition based on the cross-correlation of the time series with an assumed canonical hemodynamic response shape. All analyses reported below were based on analysis of variance (ANOVAs) and *t* tests conducted with subject as a random factor.

#### 2.3.3 Regions of interest identification

To test our hypotheses, we used a priori defined regions of interest (ROI), including left and right amygdala and insula. Voxels within a priori defined ROIs displaying group differences were identified by the repeated measure ANOVA model. To adjust for multiple comparisons two constraints were applied via AlphaSim (Ward, 2000), a voxel-wise threshold and a cluster extent (i.e. number of contiguous voxels meting the threshold). Subsequent exploratory analyses examined a much larger ROI mask, which included the left and right dorsolateral and ventral medial prefrontal cortex.

## 3 Results

### 3.1 Behavioral results

Using a 2 × 2 × 2 repeated measures ANOVA, we examined performance data in a subset of participants to determine if PTSD subjects differed in their reaction times to attending to emotional or non-emotional stimuli compared to controls as well as their overall accuracy rates. Though PTSD participants were found to be 46 ms slower on average in their response time when asked to attend to fearful faces, this difference did not meet statistical significance. No additional significant group-related differences were found in participants' reaction time or accuracy of responses. Both groups appeared to correctly identify whether pictures were the same or different approximately 80% of the time.

### 3.2 fMRI results

**Fig. 2:**
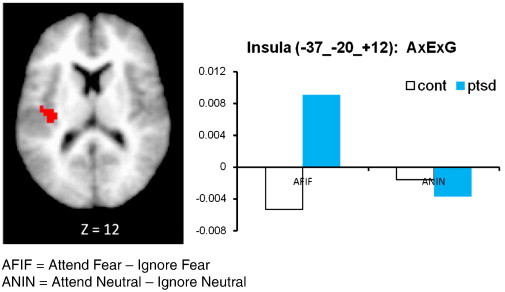
Three-way interaction of emotion and attention by group (PTSD versus healthy controls) in insula activity.

**Fig. 3:**
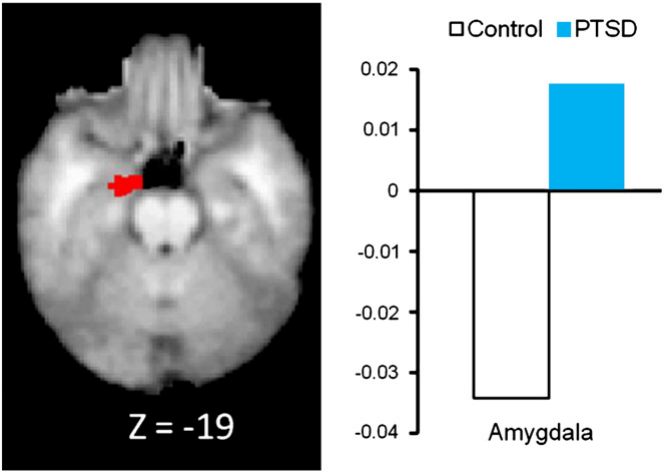
Main effect of group in amygdala activity of PTSD subjects compared with matched controls.

**Fig. 4:**
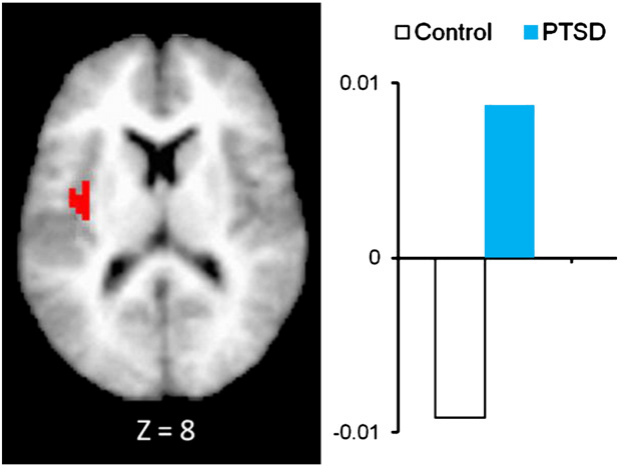
Main effect of group in insula activity of PTSD subjects compared with matched controls.

In this section we describe findings for all regions showing significant group-related effects. To test the hypothesis that PTSD participants may be more sensitive than controls to unattended fear-related stimuli in the amygdala and insula, we looked at activation in the three-way interaction of attention × emotion × group (Table 1). Examination of the left insula revealed one significant 3-way interaction between emotion × group × attention (F (1, 52) = 2.42, p = .026). PTSD had significantly higher activation in the insula when attending to fearful faces compared with ignoring fearful faces. In the control group however, participants had lower insula activation in the attend fearful faces condition (compared with ignoring fearful faces; Fig. 2). No differences were found when comparing PTSD patients and controls in the attend versus ignore neutral faces conditions. Significant main effects of group were also found for the insula and amygdala. For the left amygdala (− 12, − 10, − 19), PTSD subjects had significantly higher activation than controls (Fig. 3). PTSD subjects also had significantly higher left mid-posterior insula activity than controls (− 38, − 12, + 8; Fig. 4).

**Fig. 5:**
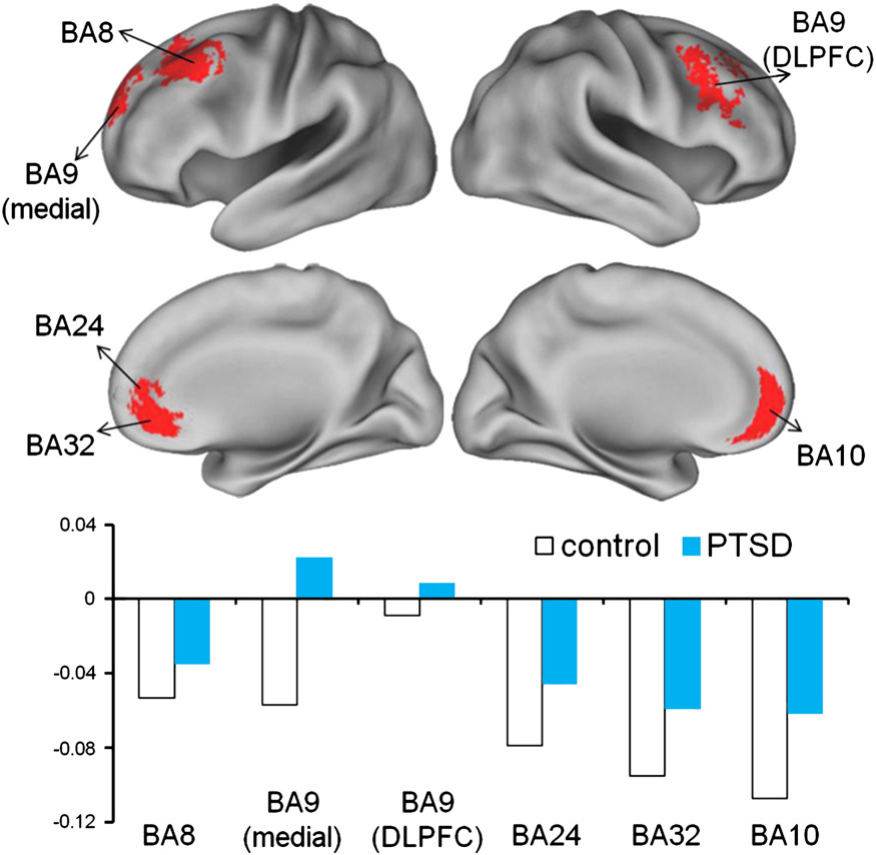
All other regions.

Upon examination of the larger exploratory ROI mask, we found several regions showing a significant main effect of group (PTSD versus controls; Fig. 5). In several areas of the left anterior cingulate and ventromedial PFC (BA24, BA10, and BA32), both groups showed reduced activation, but reductions in the PTSD group were less extensive than in the control group. Further, in one region of the left superior/medial frontal gyrus (BA9), controls showed decreasing activation, while the PTSD group showed small increases. Finally, PTSD subjects were also found to have significantly higher activation in the middle and dorsal lateral frontal gyrus (BA8 and BA9) compared with controls.

### 3.3 Correlational analyses

We conducted correlational analyses to examine the significant brain responses between PTSD and controls and specific PTSD symptom clusters (i.e. re-experiencing, numbing, hyperarousal, and avoidance). AlphaSim was used to control for inflated Type I error (Ward, 2000). In the PTSD group, it was found that total PTSD severity was significantly positively correlated with BA32 (+ 10, + 43, + 01; *p* = .042). Further, higher re-experiencing symptoms were found to be significantly positively correlated with both BA32 (+ 10, + 43, + 01; *p* = .006) and BA10 (− 01, + 04, + 07; *p* = .032). Moreover, in the ignore fear condition, insula activity was found to be significantly and positively correlated with PTSD numbing scores (− 38, − 12, + 8; *p* = .018).

## 4 Discussion

This study examined amygdala and insula activity during an emotional interference task in a sample of women with PTSD as a result of an interpersonal trauma. Our finding of left amydalar hyperactivity in PTSD subjects compared with controls adds to the literature showing increased amygdala activity in trauma-unrelated emotional tasks (Rauch et al., 2000; Shin et al., 2005; Bryant et al., 2008a, 2008b; Felmingham et al., 2010). Moreover, results from this study show increased amygdala activity during an emotional interference task, suggesting that the amygdala may play a role in implicit emotion regulation as well. However, our findings of increased amygdala activity in the PTSD group were a main effect only and not an emotion × attention interaction. Thus, it appears that heightened amygdalar activity has not been shown in this study to be specifically related to the valence of emotion as it occurs in the presence of both fearful and non-fearful stimuli. Results from this study also differed from Kim et al. (2008) as they did not find hyper amygdala activation in response to a similar paradigm. This could be due to several factors, including differences in trauma type (subway fire versus interpersonal violence), cultural differences, sample size issues, type of control group used, or possible gender effects as 7 of the 12 participants in Kim et al. (2008) were men.

With respect to the insula, results are consistent with several previous studies suggesting increased activation in the insula in PTSD subjects when presented with both trauma (Lanius et al., 2007; Lindauer et al., 2008) and non-traumatic emotional cues (Simmons et al., 2006; Fonzo et al., 2010; Strigo et al., 2010). For example, compared with healthy controls, insula hyperactivity was found in women with PTSD during the anticipation of negative versus positive images (Simmons et al., 2006). Moreover, the significant emotion × group × attention interaction found in the left insula suggests that when attending to fearful faces, PTSD subjects had significantly higher activation in the insula than did controls. These differences disappeared when neutral faces were presented. This is particularly salient as the insula has been implicated in cognition and emotional processing. These findings extend this idea and suggest that consciously attending to emotional content may further increase insular activity. The insula has also been hypothesized to serve a critical role in the development of anxiety as it may serve to generate exaggerated interoceptive cues typically found in anxiety disorders, including PTSD (Stein et al., 2007). Individuals who consciously attend to these interoceptive cues may exacerbate their anxiety symptoms thereby increasing insular activity.

Our findings differ from previous fMRI studies that have characteristically found lower activation in the dorsal lateral and ventral PFC regions. These studies have typically used trauma cues such as traumatic script-driven imagery (Shin et al., 1999; Lanius et al., 2001; Shin et al., 2004). For example, in a sample of sexual abuse and motor vehicle accident victims, Lanius et al. (2001) found decreased medial frontal gyrus and rostral ACC activation in a script-driven symptom provocation paradigm compared with healthy, traumatized controls without PTSD. Decreased vmPFC and ACC activation has also been in a few studies using fearful faces unrelated to the trauma (Shin et al., 2005; Williams et al., 2006; Kim et al., 2008). In particular, Kim et al. (2008) found decreased rostral ACC activity when presented with a similar emotional processing conflict task as the current study, suggesting that the rostral ACC may play an important role in the development of PTSD.

However, more recent studies have been consistent with results from the current study that found greater mPFC activity and less suppression of dACC activity in a PTSD sample (Shin et al., 2007; Bryant et al., 2008a, 2008b; Fonzo et al., 2010; Fani et al., 2012). In a sample of fifteen patients with PTSD, Bryant et al. (2008a, 2008b) reported significantly higher mPFC activation than non-traumatized healthy controls when presented with unconsciously processed fearful versus neutral faces, providing evidence to suggest that elevated mPFC activity may be related to nonconscious fear processing. Moreover, in an attention bias task, Fani et al. (2012) found that compared to healthy trauma-exposed controls, PTSD participants exhibited higher DLPFC activation when exposed to threat cues as well as greater dACC activity during attentional avoidance of threat. Our results may extend this interpretation further to include the notion that implicit emotional regulation may also increase medial and dorsal lateral PFC activity in PTSD patients. However, it should be noted that sample characteristics of both the study group and comparison group might account for differences across studies. For example, greater severity or higher co-morbidity of the study group and healthy versus trauma exposed comparison groups both could account for inconsistent results. Moreover, incongruent results with the Kim et al. (2008) study (which used a similar emotional conflict paradigm) may be due to a variety of factors as mentioned earlier including the authors hypothesize that responses to fearful faces may not be as strong in individuals with an Asian cultural background (Kim et al., 2008).

It has been previously hypothesized that over activity of the amygdala in PTSD patients was due in part to, a failure of top-down control in which hypoactivity of the dlPFC and ACC failed to regulate amygdalar activity (Shin et al., 2006; Kim et al., 2008; Simmons et al., 2011). Our results do not support this hypothesis for several potential reasons. First, the task used in this study was an implicit emotional regulation paradigm that involved cognitive decision making. This is consistent with recent studies (Moores et al., 2008; Daniels et al., 2010) that attending to working memory tasks demand a greater effort in patients with PTSD. Thus, PTSD subjects may engage in compensatory recruitment of brain networks which may be over active during task performance. Therefore, differing results may be attributed to the particular task conducted in the scanner. Secondly, a recent examination of top-down control suggests that it may not be a single entity. It has been suggested that a dual-networks model of control is a more accurate representation during a cognitive task, with top-down control being driven by at least two separate control networks that are distributed throughout multiple brain regions, including the prefrontal cortex and insula (Dosenbach et al., 2008).

Results from this study suggest that re-experiencing symptoms were significantly correlated with activity level in some default mode network (DMN) areas. The DMN is a group of brain regions characterized by decreased activity when a healthy individual is engaged in goal-directed behavior (Shulman et al., 1997). Reduction of activity in the DMN during effortful cognitive processing can be interpreted as reflecting the need to attenuate the brain's self-referential activity as a means of more effectively focusing on a task. Our findings suggest that in individuals with PTSD who have higher re-experiencing symptoms, the DMN exhibited altered activity. For example, in several DMN areas (ventromedial prefrontal cortex prefrontal cortex (BA 10), anterior cingulate (BA 32), PTSD subjects exhibited less of a reduction of activity during the emotional conflict task. These data suggest PTSD is characterized by stimulus-induced heightened activity and a failure to normally down-regulate activity broadly within the DMN (Sheline et al., 2009). In addition, the insula, which falls within the cingulo-opercular network and the DLPFC, which falls within the fronto-parietal network, also exhibited increased activity during implicit emotional regulation. In the insula, increased activation to the ignore fear condition was correlated with the magnitude of emotional numbing. This may suggest that unconscious implicit emotional activation to fear occurs to a greater degree in patients with greater shutting down of emotional receptivity, given the involvement of the insula in interoception and anticipatory anxiety (Schunck et al., 2008; Lovero et al., 2009).

Given that the PTSD sample was composed of only women who have experienced at least one interpersonal trauma, results may not generalize to men or to other types of traumas. Further, the absence of a trauma-exposed no-PTSD group limits our ability to delineate the influence of trauma exposure on neural networks. However, our findings suggest the presence of several brain abnormalities in women with PTSD. Importantly, the specific emotional conflict task used appears to target implicit or automatic emotional regulation instead of explicit or effortful emotional regulation. This is particularly relevant as it is posited that emotional regulatory difficulties in anxiety disorders such as PTSD appear to occur in implicit forms of emotion regulation (Etkin, 2010).

## Figures and Tables

**Table 1:** Group-related effects for the conflict task.

Brain region	BA	Voxels	Side	Talairach coordinates			Z-val.	Effect
				x	Y	z		
*Attention × emotion × group (× time)*								
Insula^a^	13	23	L	− 37	− 20	12	2.22	PTSD > Cont

*Main effect of group (PTSD versus controls)*								
Amygdala^b^	34	17	L	− 12	− 10	− 19	3.60	PTSD > Cont
Insula^a^	13	59	L	− 38	− 12	8	2.29	PTSD > Cont

Main effect of group (PTSD versus controls)								

Brain region	BA	Voxels	Side	Talairach coordinates			Z-val.	Effect

X	y	Z

^c^Anterior cingulate	24	27	L	0	33	9	3.07	PTSD > Cont
^c^Anterior cingulate	32	428	L	− 1	47	7	4.67	PTSD > Cont
^c^Anterior cingulate	32	94	R	10	43	1	3.60	PTSD > Cont
^c^Medial frontal gyrus	10	117	L	− 11	45	2	3.39	PTSD > Cont
^c^Medial frontal gyrus	10	30	R	1	59	− 4	3.23	PTSD > Cont
^c^Superior/medial frontal gyrus	9	76	L	− 18	53	31	3.66	PTSD > Cont
^c^Middle frontal gyrus (lateral)	9	71	R	34	17	31	4.12	PTSD > Cont
^c^Middle frontal gyrus	8	95	R	30	23	45	3.78	PTSD > Cont

Brain regions showing group difference in the pattern of task-based activity time series between PTSD (n = 32) and control (n = 21): ^a^Insula mask only; ^b^amygdala mask only; ^c^all-ROIs mask.
